# Ex vivo RSA and Pfkelch13 targeted-amplicon deep sequencing reveal parasites susceptibility to artemisinin in Senegal, 2017

**DOI:** 10.21203/rs.3.rs-2538775/v1

**Published:** 2023-02-06

**Authors:** Mamadou Samb Yade, Baba Dièye, Romain Coppée, Aminata Mbaye, Mamadou Alpha Diallo, Khadim Diongue, Justine Bailly, Atikatou Mama, Awa Fall, Alphonse Birane Thiaw, Ibrahima Mbaye Ndiaye, Tolla Ndiaye, Amy Gaye, Abdoulaye Tine, Younouss Diédhiou, Amadou Mactar Mbaye, Cécile Doderer-Lang, Mamane Nassirou Garba, Amy Kristine Bei, Didier Ménard, Daouda Ndiaye

**Affiliations:** Centre International de Recherche et de Formation en Génomique Appliquée, et de Surveillance Sanitaire (CIGASS), Cheikh Anta Diop University of Dakar; Centre International de Recherche et de Formation en Génomique Appliquée, et de Surveillance Sanitaire (CIGASS), Cheikh Anta Diop University of Dakar; Université Paris Cité and Sorbone Paris Nord, Inserm, IAME; Centre for Research and Training in Infectiology of Guinea (CRTIG); Centre International de Recherche et de Formation en Génomique Appliquée, et de Surveillance Sanitaire (CIGASS), Cheikh Anta Diop University of Dakar; Cheikh Anta Diop University of Dakar; University of Paris Cité, IRD, MERIT; Université de Paris, Institut Cochin, Inserm U1016; Centre International de Recherche et de Formation en Génomique Appliquée, et de Surveillance Sanitaire (CIGASS), Cheikh Anta Diop University of Dakar; Department of Biochemistry and Functional Genomics, Faculty of Medicine and Health Sciences; Centre International de Recherche et de Formation en Génomique Appliquée, et de Surveillance Sanitaire (CIGASS), Cheikh Anta Diop University of Dakar; Centre International de Recherche et de Formation en Génomique Appliquée, et de Surveillance Sanitaire (CIGASS), Cheikh Anta Diop University of Dakar; Centre International de Recherche et de Formation en Génomique Appliquée, et de Surveillance Sanitaire (CIGASS), Cheikh Anta Diop University of Dakar; Centre International de Recherche et de Formation en Génomique Appliquée, et de Surveillance Sanitaire (CIGASS), Cheikh Anta Diop University of Dakar; Centre International de Recherche et de Formation en Génomique Appliquée, et de Surveillance Sanitaire (CIGASS), Cheikh Anta Diop University of Dakar; Centre International de Recherche et de Formation en Génomique Appliquée, et de Surveillance Sanitaire (CIGASS), Cheikh Anta Diop University of Dakar; Université de Strasbourg, UR7292 Dynamics of Host-Pathogen Interactions; Centre International de Recherche et de Formation en Génomique Appliquée, et de Surveillance Sanitaire (CIGASS), Cheikh Anta Diop University of Dakar; Yale School of Public Health; Université de Strasbourg, UR7292 Dynamics of Host-Pathogen Interactions; Centre International de Recherche et de Formation en Génomique Appliquée, et de Surveillance Sanitaire (CIGASS), Cheikh Anta Diop University of Dakar

**Keywords:** Malaria, Plasmodium falciparum, Artemisinin partial resistance, Ring-stage Survival Assay, Pfkelch13 genotype, targeted-amplicon deep sequencing, Senegal

## Abstract

**Introduction.:**

Malaria control is highly dependent on the effectiveness of artemisinin-based combination therapies (ACTs), the current frontline malaria curative treatments. Unfortunately, the emergence and spread of parasites resistant to artemisinin (ART) derivatives in Southeast Asia and South America, and more recently in Rwanda and Uganda (East Africa), compromise their long-term use in Sub-Saharan Africa where most malaria deaths occur.

**Methods.:**

Here, we evaluated *ex vivo* susceptibility to dihydroartemisinin (DHA) from 38 *P. falciparum* isolates collected in 2017 in Thiès (Senegal) expressed with the Ring-stage Survival Assay (RSA). We explored major and minor variants in the full *Pfkelch13* gene, the main determinant of ART resistance using a targeted-amplicon deep sequencing (TADS) approach.

**Results.:**

All samples tested in the *ex vivo* RSA were found to be susceptible to DHA. Both non-synonymous mutations K189T and K248R were observed each in one isolate, as major (99%) or minor (5%) variants, respectively.

**Conclusion.:**

Altogether, investigations combining *ex vivo* RSA and TADS are a useful approach for monitoring ART resistance in Africa.

## Background

Prompt management of malaria cases remains a vital component of malaria control and elimination strategies [1]. Over the two last decades, artemisinin (ART)-based combination therapies (ACTs) have contributed significantly to the reduction of malaria-related morbidity and mortality in Sub-Saharan Africa[2]. Since 2006, the Senegalese National Malaria Control Program (NMCP) have recommended artemether–lumefantrine (AL) as the first-line treatment for uncomplicated malaria [3]. However, recent reports of the emergence and expansion of partial resistance to ART (ART-R) in the Greater Mekong Subregion have endangered the long-term efficacy of ACTs. Although ACTs remain clinically and parasitologically effective in most African malaria endemic countries [4], the local emergences of ART-R in Rwanda and in Uganda, respectively reported in 2020 and 2021, are warning signals in the loss of ART efficiency [5–8]. As ART-R could independently emerge in western Africa or spread from eastern African ATR-R hotspots, the monitoring of *P. falciparum* parasites susceptibility to ART-based on the current clinical and biological tools is of utmost importance and must be implemented [4]. Clinically, ART-R is defined as delayed parasite clearance or Day-3 positive parasitemia upon ART-based treatment [9]. Delayed clearance has been associated with decreased *in vitro* parasite susceptibility (survival rate ≥1%) expressed in the Ring-stage Survival Assay (RSA^0–3h^) [10]. These clinical and biological phenotypes were later associated with non-synonymous mutations in the *P. falciparum kelch13* gene (*Pfkelch13*) [11]. *Pfkelch13* encodes a 726-amino acid protein containing three structurally conserved domains: a coiled-coil-like (CCC) domain, a Broad-Complex, Tramtrack and Bric a brac (BTB) domain, and a C-terminal kelch-repeat propeller domain where most of non-synonymous mutations associated with ART-R have been described [12]. Since then, the surveillance based on the detection of *Pfkelch13* single-nucleotide polymorphisms (SNPs) have been conducted in many countries. In the Greater Mekong Subregion, multiple ART-R *Pfkelch13* mutant parasites evolved concomitantly until a multidrug-resistant *Pfkelch13* C580Y lineage (named *KEL1/PLA1*) outcompeted the others and spread across Southeast Asia [13]. In Africa, this variant has been sporadically reported [14–16]. Rather, the *Pfkelch13* R561H mutant rapidly increased in frequency in Rwanda between 2014–2016 and 2018–2019 (from 8–22%, respectively) [17–19] and was associated with *in vivo* and *in vitro* ART-R [19]. Similarly, in Uganda, two *Pfkelch13* mutants (A675V and C469Y) were recently reported to be associated with *in vivo* and *in vitro* ART-R [6, 8, 20]. In Senegal, most investigations have looked for SNPs in the propeller-encoding domain of *Pfkelch13* gene using conventional methods such as PCR/Sanger sequencing [21–26], except for two studies that used *Pfkelch13* targeted-amplicon deep sequencing (TADS) [25, 26]. Although the authors did not detect validated or candidate ART-R *Pfkelch13* mutations [27], TADS approach is particularly relevant to detect the presence of minor variants in *P. falciparum* isolates. Here, *Pfkelch13* genotype was evaluated by TADS in 38 *P. falciparum* isolates collected in Thiès in 2017. We also explored the genetic variation of the whole conserved-domains of *Pfkelch13* gene owing that mutation in the BTB-encoding domain of *Pfkelch13* gene in a western African strain has been shown to be associated with ART-R [28].

## Materials And Methods

### Ethics.

The National Ethics Committee for Health Research and the Ministry of Health of Senegal approved the protocol used for this study under number 00000169/MSAS/DPRS/CNERS (December 2nd, 2016). Written and informed consent was obtained from all participants, before participant recruitment and sample collection.

### Study site.

The study was conducted during the peak malaria season (September to December) in 2017 in Thiès (Senegal; 14° 47′ 26″ north, 16° 55′ 29″ west), an area belonging to the Sahelian facies defined by a short malaria seasonal transmission (< 4 months). In this region, the entomological inoculation rate (EIR) is low, estimated to be < 5 [29], and malaria is mainly transmitted by *Anopheles arabiensis* and *Anopheles gambiae* mosquito vectors.

### Study design.

Individuals who visited the Service de Lutte Antipaludique (SLAP) clinic with signs and symptoms suggestive of uncomplicated *P. falciparum* malaria were screened by microscopic examination on Giemsa-stained blood smears. Malaria infected patients were treated with artemether-lumefantrine (AL, Coartem), according to the treatment guideline of the Senegal National Malaria Control Program (NMCP). For each patient, 5 mL vacutainer tubes of venous blood were collected for *ex vivo* RSA.

Parasite culture and ring-stage survival assay. Parasitemia was estimated by microscopy examination on Giemsa-stained blood smears. Venous blood samples were then processed by eliminating plasma, leukocytes and anticoagulant from red blood cells (RBCs), washed twice in RPMI 1640 medium (Gibco, Life technologies). Parasitemia were adjusted to 1% if greater by adding uninfected RBCs as previously described [10]. Then, 900 μL RBCs were loaded into wells, exposed to either 100 μL of 700 nM DHA or0.1% of dimethyl sulfoxide (DMSO) and cultivated at 37°C in incubator for six hours (conditions: 94% N_2_, 5% CO_2_, 1% O_2_) [30]. Finally, RBCs were washed and cultivated for 66 hours. The proportions of viable parasites were estimated independently by two expert malaria microscopists on Giemsa-stained thin smears. The number of viable parasites that developed into ring/trophozoite stages were determined, pyknotic forms were excluded. The average of the two counts was calculated. If any discrepancy was noted (either difference of parasite density of > 50%), slides were checked by a third independent reader, and parasite densities were calculated by averaging the two most close counts. Survival rates were calculated as the ratio of parasites in exposed and non-exposed cultures. Results were deemed as interpretable if the parasitemia in the sample exposed to DMSO was higher than the initial parasitemia at 0h [31].

### DNA extraction.

DNA was extracted from the same whole blood sample used for the *ex vivo* RSA using the QIAamp DNA Blood Mini Kit (Qiagen, Valencia, CA, USA) according to manufacturer instructions.

### Targeted-amplicon deep sequencing of Pfkelch13.

Extracted DNA samples were subjected to two separate overlapping PCRs covering the three conserved-encoding domains of the *Pfkelch13* gene (one fragment covering the CCC and BTB domains: 5’-agatgcagcaaatctta-3’ and 5’-ttctacaccatcaaatcc-3’; and the second fragment covering from the end of the CCC domain to the propeller domain (5’-aaaaagaaaaagaagaacataggaaa-3’and 5’-tgtgcatgaaaataaatattaaagaag-3’). Briefly, 1 μL of DNA was amplified with 0.25 μM of each primer, 0.2 mM of dNTP, 0.625 unit of GoTaq G2 Flexi DNA Polymerase (Promega, Madison, USA) and 2.5 mM and 3 mM of MgCl2 for the first and the second fragment respectively. The cycling conditions were as follows: 3 min at 95°C, then 40 cycles of 30 s at 95°C, 30 s at 55°C, 90 s at 68°C and final extension 3 min at 68°C. PCR products were detected using 1% agarose gel electrophoresis and SYBR Safe staining (Invitrogen, Waltham, USA). DNA from *P. falciparum* 3D7 strain and water were used as positive and negative controls, respectively. Generated amplicons were then sequenced. Briefly, PCR amplicons were fragmented with the Covaris S220 to about 150 bp, and libraries were constructed using the KAPA hyper Prep Library Preparation Kit (Kapa Biosystems, Woburn, MA) following the manufacturer’s protocol. Amplicons were purified and the library size selection was performed with AMPure Agencourt XPbeads. Libraries quality and quantity control were assessed using Qubit^®^ for concentration and Bio Analyser 2100 Agilent for fragment size. Libraries were pooled at approximately equal concentration and sequenced on an Illumina NextSeq 500 instrument (Illumina Inc, San Diego, CA, USA) to generate 150 bp paired-end reads at the GENOM’IC platform of Cochin Institute (Paris, France). Raw sequences were then demultiplexed and quality trimmed at a Phred score of 30. Primer sequences were trimmed from the 5′-end of the sequences to avoid primer bias in the sequenced fragments. Base calling was performed by comparing reads with a custom database consisting of the *Pfkelch13* sequence retrieved from the 3D7 reference genome. Bioinformatic analyses were performed using the CLC Genomics Workbench 22 software (Qiagen).

## Results

### Baseline characteristics.

A total of 38 patients with uncomplicated *P. falciparum* malaria meeting the inclusion criteria were enrolled. The sex ratio (M/F) was largely dominated by males (36 males/2 females). The age of the participants ranged from 9 to 70 years (median of 21.5 years). Median weight was 59.5 kg and median body temperature was 38.5°C. The median parasitemia was 1.03%, ranging from 0.68–1.53% (Table 1).

### Ex vivo RSA phenotype.

Examination of blood smears and species-specific PCR revealed that all samples were pure *P. falciparum* infections. *Ex vivo* RSA were successfully performed for the 38 tested samples. Survival rates showed the absence of surviving parasite to the 700nM DHA pulse as for the 3D7 strain (0%) except for three isolates Th54, Th77 and Th95 (0.054%, 0.06% and 0.22% respectively) which were however less than the 1% threshold.

### Pfkelch13 genotyping.

Among the 38 samples successfully sequenced, two non-synonymous mutations (K189T and K248R) located outside of the propeller-encoding domain were detected (Table 2). Each mutation was observed in a single sample in major (99% for K189T) and minor (5% for K248R) proportions. The *Pfkelch13* K248R mutant was detected for the first time in Senegal (Table 3).

## Discussion

ACTs are now the mainstay of treatment for uncomplicated malaria in malaria endemic regions [2]. Unfortunately, the emergence and the clonal expansion of *Pfkelch13* mutant parasites have been reported recently in Rwanda (R561H) and Uganda (C469Y and A675V) [5–8]. Therefore, the World Health Organization (WHO) recommends to closely monitor the susceptibility of *P. falciparum* to antimalarial drugs and particularly to ART derivatives [4]. As in many Sub-Saharan African countries, ACTs have contributed significantly to the decline in malaria incidence and mortality over the past decade. In Senegal, considerable efforts have been done to reduce malaria morbidity and mortality mainly since 2006 when AL was introduced [3]. Consequently, the emergence of ART-resistant parasites is a major threat which can hinder road to malaria elimination.

Partial resistance to ART is associated with non-synonymous mutations in the *Pfkelch13* gene. However, the impact of most mutations on ART-R detected in field samples is largely unknown mainly due to the lack of association between the clinical phenotype (delayed parasite clearance) and the *Pfkelch13* genotype. The study presented here aims to fill this gap by combining *ex vivo* Ring-stage Survival Assay (RSA) with *Pfkelch13* genotyping. The *ex vivo* RSA estimates the susceptibility of *P. falciparum* to ART using parasite isolates freshly collected from patients with malaria. The *ex vivo* RSA has been previously used in studies conducted in Central [32] and East Africa [31, 33]. Four isolates from Uganda showed high (> 10%) survival rates, levels of which are reported to be closely associated with delayed parasite clearance following artesunate monotherapy [31]. The data presented here showed that none of the tested samples confer *in vitro* ART-R with a survival rate ≥ 1%, suggesting the absence of decrease susceptibility of Senegalese parasites to ART derivatives.

Partial resistance to ART has also been associated with specific mutations in the *Pfkelch13* gene [1, 2]. Since 2015, this molecular marker has been extensively used to seek *Pfkelch13* mutants in Sub-Saharan African countries. By using a targeted amplicon deep sequencing approach, we detected here two mutations (K189T and K248R). The K248R mutation was observed in one sample in minor proportion (5%). The mutation is located in the CCC domain of *Pfkelch13*, but we think the mutation not likely related to ART-R since the survival rate was less than 1%. While K189T had already been reported in Africa [34,35], the non-synonymous mutation K248R was detected for the first time in Senegal. The isolate carrying the K189T mutation had also a survival rate < 1%, confirming that this allele does not confer *in vitro* ART-R as previously observed in India [36]. Only three *Pfkelch13* wild-type isolates (Th54,Th77 and Th95) were found to have a survival rate above 0%. To date, *in vitro* susceptibility to ART derivatives by RSA of *P. falciparum* isolates have been reported in Cameroon and Uganda. Two studies conducted in Cameroon [32, 33] showed the absence of *Pfkelch13* mutations associated with ART-R while one study in Uganda[31] reported high survival rates of isolates carrying *Pfkelch13* A675V and C469Y mutations.

The study presented here has several limitations. First, only 38 samples were tested owing that *ex vivo* RSA is time consuming and requires skilled personnel. Second, *in vitro* RSA (from culture-adapted parasites) was not performed to confirm the *ex vivo* survival rates. And third, the study was conducted only in one site in Senegal (Thiès) and no clinical data on delayed parasite clearance (like Day-3 positivity rate) was collected.

## Conclusion

This study shows that combining *ex vivo* RSA phenotype and *Pfkelch13* genotyping can be efficiently carried out to monitor ART-R and providing relevant data to malaria control programs on parasite susceptibility to ART. Particularly, targeted-amplicon deep sequencing used here confirmed to be useful to detect the presence of minor alleles. This study showed that all *P. falciparum* isolates collected in Thiès were susceptible to DHA. As recommended by the WHO [4], similar studies must be conducted in Senegal and other African countries to strengthen surveillance of antimalarial drug efficacy and resistance and minimize the threat and impact of antimalarial drug resistance in Africa.

## Figures and Tables

**Table 1:** Baseline characteristics of the study participants and the *P. falciparum* isolates, Thies, Senegal, 2017.

	median	CI95%	IQR25–75
Age, years	21.5	17.0 to 25.0	15.0–28.0
Weight, kilogram	59.5	50.5 to 64.5	38.0–67.0
Temperature, °C	38.5	38.2 to 39.1	37.8–39.5
Glycemia	0.9	0.9 to 10.9	0.8–1.0
Hemoglobin	13.4	12.1 to 14.2	11.5–15.4
Parasitaemia %	1.0	0.7 to 1.5	0.6–1.9

CI95%: Confidence interval 95%; IQR: Interquartile range

**Table 2:** Proportion of non-synonymous *Pfkelch13* mutations detected in the 38 *P. falciparum* isolates

Codon position	Reference sequence		Mutant sequence		Type	n/N
	Amino acid	Nucleotide	Amino acid	Nucleotide		
189	K	AAA	T	ACA	NS	1/38
248	K	AAG	R	AGG	NS	1/38

Note: The boldface highlights the nucleotide base change. Abbreviations: n, number of samples containing mutant allele; N, total number of successfully sequenced samples; NS, non-synonymous mutation

**Table 3:** List of the mutations in the *Pfkelch13* gene previously observed in Senegal and in the present study

Plasmodium-specific/CCC/BTB-POZ	Propeller	Authors	Year(s)	Year(s)
T149S and K189T* N142N/NN (insertion)	No mutations	Madamet et al.	2012–2013	Dakar
D109H, L119L, H136N, P152P, M163I, K189T*, K189N, D214G, M235I, R239L, R255K, L258M, K278N, G287C, K293N, I313I, G357V, T367T, L368I, R398M, S423I, L429F	P443Q, S459S, Q468Q, C469C, C473F, W518C, G533V, R539I, R553I, V555L, E556V, P570L, A578D, R587I, G592V, R597I, A621A, A626V, W660C, G690G	Talundzic et al.	2011–2014	Dakar and Thiès
K123R, N137S, N142NN/NNN, T149S, and K189T/N	N554H, Q613H, and V637I	Boussaroque et al.	2013–2014	Dakar
D109H, T149S, K189N, K189T*, H274Y, D283Y, D389Y, N141-N142NN, N142N	T478T, A578S and V637I	Gaye et al.	2015–2016	Dakar (2015) Kédougou and Matam (2016)
No mutations	C469C, F491F, G545G, F583F and A627A	Ahouidi et al.	2015, 2016 and 2017	Bounkiling
No mutations	No mutations	Diallo et al.	2018	Diourbel and Kédougou
No mutations	No mutations	Delandre et al	2015, 2016, 2017, 2018 and 2019	Dakar
K189T*, K248R	No mutations	This Study	2017	Thiès

* Most frequent mutations

## Data Availability

The data that support the findings of this study are available from the corresponding author on reasonable request. All relevant data are within the manuscript.

## Abbreviations

ACT: Artemisinin-based combination therapy
RSA: Ring-stage Survival Assay
TADS: Targeted-amplicon deep sequencing
*Pfkelch13*
PCR: Polymerase chain reaction
DNA: Deoxyribonucleic acid
NMCP: National Malaria Control Program
WHO: World Health Organization
